# Sadness and Other Health Complaints among Swedish Adolescents: A Cross-Sectional Study

**DOI:** 10.3390/ijerph18083999

**Published:** 2021-04-10

**Authors:** Tide Garnow, Eva-Lena Einberg, Anna-Karin Edberg, Pernilla Garmy

**Affiliations:** 1 Faculty of Health Sciences, Kristianstad University, SE-291 88 Kristianstad, Sweden; evalena.einberg@hkr.se (E.-L.E.); anna-karin.edberg@hkr.se (A.-K.E.); pernilla.garmy@med.lu.se (P.G.); 2 WHO-CC (World Health Organization Collaborative Centre), Clinical Health Promotion Centre, Lund University, SE-205 02 Malmö, Sweden

**Keywords:** adolescents, sadness, health complaints, embodied emotions, a cross-sectional study

## Abstract

Health complaints are increasing among adolescents and are recognized as a global public health issue. Health complaints are an indicator for subjective ill-being, but little is known about the relationship between sadness and other health complaints. The aim of this study was to investigate sadness and other health complaints among Swedish adolescents. A survey with a cross-sectional design was completed by adolescents (*n* = 1489, 15–17 years old) in the south of Sweden. A logistic regression analysis was used to analyze the relationship between sadness and other health complaints. The result show that sadness and other health complaints were common among adolescents, and sadness was related to health complaints (headache (OR: 1.58), sleeping difficulties (OR: 2.00), reduced appetite (OR: 1.43), tension (OR: 2.44), and concentration difficulties (OR: 2.75)). When adolescents express sadness or other health complaints it is important to reflect on what these complaints are an expression of, and take into account the body as physical and psychological intertwined. This might entail person-centered support that hopefully leads to an improvement in adolescents’ well-being. Future research that profoundly highlights adolescents’ existential health is needed.

## 1. Introduction

Adolescents generally rate their health as good; nevertheless, health complaints are common among adolescents [1] and are now recognized as a global public health issue [2]. Health complaints consist of the subjective experience of psychological and physical symptoms, such as tension, headache, and insomnia, and can be viewed as an indicator for present subjective ill-being [3]. Health complaints among adolescents have been shown to be associated with medical as well as psychological, social, and health behavior factors [4]. Health complaints can thus be interpreted in various ways and it can be challenging to provide appropriate support. Additionally, sadness is a common emotion that repeatedly occurs during life, but sadness is also one of the main symptoms of depression. A large body of previous research has examined depression among adolescents [5], but little is known about nonpathological sadness [6]. We know little about adolescents’ feelings of sadness in particular and the relationship between sadness and other health complaints.

### 1.1. Health Complaints among Adolescents

Studies show that the prevalence of health complaints has increased during recent decades, particularly among female adolescents [7]. Among researchers there are different interpretations of these trends. Possible explanations are rising affluence and worsening income inequality [8], a more vulnerable labor market [9], and the negative impact of individualization processes [10]. Another explanation may be that adolescents have been viewed as a healthy group with low mortality for a long time, and therefore their health has not been a great priority [2]. Other possible explanations are described as difficulties in the family environment, increasing pressure within school [11], and increasing screen time [12]. Additionally, the relationships between health complaints among adolescents and psychosocial factors are numerous. Research has shown that there is an association between health complaints and being a female adolescent, experiencing school-related pressure [13], having low socio-economic status [14], being victimized by harassments and bullying [15], and having strained relationships with parents and peers [16]. Health complaints among adolescents can thus be interpreted as expressions of stressful life events.

### 1.2. Developmental Changes during Adolescence

Adolescence is often described as chaotic [17] and includes many developmental challenges, such as accommodating physical changes, developing an identity, and dealing with changing interpersonal relationships. According to Erik H. Erikson’s theory, Stages of Psychosocial Development, adolescence extends between the ages of 12 and 20 years and is a phase where children approach adulthood and create their own identity [18]. Adolescence involves conflicting demands and uncertainty for the future, and the phase additionally coincides with pubertal body development. Erikson argues that this can create role confusion and identity crisis. Adolescence is thus a period of great transition and the emotional experiences are subject to significant changes [19]. Positive emotions decrease during adolescence, while negative emotions increase [20]. However, this emotional chaos is not always clearly outspoken and may thus be difficult to detect or understand. Negative emotions can take on a physical expression. It has been shown that sadness can be expressed through, for instance, insomnia and pain [3]. A qualitative study that explored mothers’ understanding of their children’s medically unexplained physical symptoms showed that mothers sometimes interpreted the causes as psychological or emotional [21]. One way of interpreting the relationship between sadness and physical expressions is by describing sadness as an emotion being embodied.

### 1.3. Theoretical Background

Concepts of embodiment have gained increasing importance in research in recent decades [22]. Embodiment can be understood through neuroscience, as well as through social psychology and philosophy. According to the French philosopher and existentialist Maurice Merleau-Ponty [23], the body is an expressive space and the center of experiences. Humans perceive everything through the body, and the physical expressions point out the relationship between the human being and her world of mind and emotions. Merleau-Ponty even argues that the perceptions of phenomena are a bodily function. It is through the body that consciousness is formed, and therefore the inner emotion and the outer expression cannot be divided. Emotions occur in relation to others and the world and are expressed in our bodies. The psychiatrist and philosopher Thomas Fuchs [22] has further developed Merleau-Ponty’s ideas of embodiment. Fuchs addresses it as an “embodied subjectivity”, where the person is a unity of the subjective “lived body” and the “physical body”. Those two units cannot be separated, and there is a circular interaction of psychological and biological processes. Neither mental well-being nor mental ill-being is limited to the brain.

Since emotions are expressed in the body, it can be a challenge to understand what health complaints stand for and also how they can be responded to. When adolescents address health complaints, it is thus important that they are offered person-centered support to promote their well-being. By investigating sadness and other health complaints among adolescents, we will hopefully create knowledge that leads to a positive impact on adolescents’ well-being, both in the present and in the later adulthood.

### 1.4. Aim

The aim of this study was to investigate sadness and other health complaints (sleeping difficulties, headache, abdominal pain, reduced appetite, tension and concentration difficulties) among Swedish adolescents.

## 2. Materials and Methods

### 2.1. Study Design

The study employed a quantitative design and was performed as a cross-sectional study based on survey data. The study was approved by the Regional Ethics Review Board in Lund, Sweden (EPN 2017/600). All procedures were conducted in accordance with the Declaration of Helsinki.

### 2.2. Context

The survey was conducted in a municipality in the south of Sweden. According to EU Statistics on income and living conditions, Sweden has the lowest share of material poverty in Europe [24]. In addition, the municipality is among the municipalities in Sweden with the most highly educated residents, according to Swedish national statistics [25]. In Sweden, compulsory schooling lasts for 9 years in elementary school. This is followed by a 3-year upper secondary education, which is not compulsory. However, according to national statistics, only half a percent of Sweden’s adolescents do not enter upper secondary education [26]. At upper secondary school, there are both vocational programs and university preparatory programs. In Sweden, the citizens are offered free education, even at the university level, and according to national statistics from 2019, as many as 44% of the residents in Sweden have attended higher education [27]. In Sweden more women than men acquire a higher education (50% vs. 38%).

### 2.3. Sample and Data Collection

**Figure 1:**
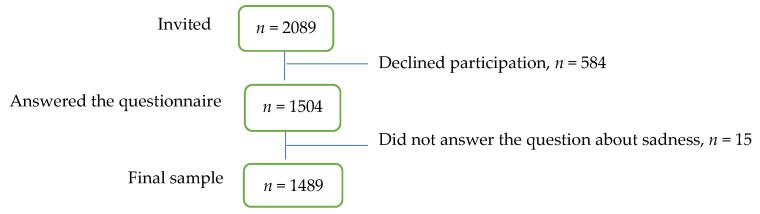
Flow-chart for the selection process of the study participants.

The total sample consisted of 2089 first-year students in upper secondary school (15–17 years old) in a municipality in the south of Sweden. Five different upper secondary schools were asked to participate in the study: three public schools and one private school accepted. The students came from both rural and urban areas. The aim was to have a sample size more than 10 times the number of included variables [28]. 1504 students agreed to participate and answered the questionnaire; however, 15 students did not respond to the question about sadness. Therefore, the final sample comprised 1489 students (71.3% response rate) (Figure 1). Of the participants, 55.4% were female adolescents, 43.7% were male adolescents, while 1.7% answered “other” which indicates nonbinary identity [29]. The majority (*n* = 1412) of the students attended university preparatory programs, and the remaining students (*n* = 77) attended vocational programs. There was no statistically significant difference in sadness between the students in the vocational and the university preparatory programs (*p* = 0.470).

The data were collected between October 2017 and May 2019 by a web survey. The survey was distributed during school hours to all first-year students. The students and their parents or legal guardians were informed in writing about the purpose of the study. The students’ participation was voluntary, and the declining students were not asked why they did not want to participate. Reasons for dropout were that students were absent on the day of the survey distribution or that they declined to participate.

The survey included questions about the participants’ gender, perceived family financial situation, sadness, sleeping difficulties, headache, abdominal pain, reduced appetite, tension, and concentration difficulties (see Appendix A). The questions used in this study have been shown to be common health complaints among adolescents in previous research. The questions are derived from the Health Behaviour in School-Aged Children (HBSC) survey, which is a collaborative cross-national project conducted by World Health Organization [1]. The questionnaire has been used since the 1980s and is tested for reliability and validity [30].

### 2.4. Data Analysis

Descriptive statistics with frequencies (*n*) and percentages (%) were used to present the data. A bivariate analysis (Pearson’s chi-squared tests) was used to test the hypothesis of a probable relationship between the dependent variable sadness and the different independent variables. All the variables were dichotomized to acquire a clearer picture of the material; some answer options had few cases and without a dichotomization, it would have been difficult to interpret the result. The variables used in the analysis were: sadness, sleeping difficulties, headache, abdominal pain, reduced appetite, tension, concentration difficulties, perceived family financial situation, and gender. Response options and coding are presented in Appendix A.

Binary logistic regression was used to analyze the confidence interval (CI), and the odds ratio (OR) was used to represent the data. In the crude analysis, the relationship between sadness (often or always sad) and other health complaints (sleeping difficulties, headache, abdominal pain, reduced appetite, tension and concentrations difficulties) was investigated. Health complaints were found to be affected by gender and socio-economic status in earlier studies [7,13,14]; therefore, we adjusted for this in the multivariate binary logistic regression model.

The confidence interval was calculated at 95%. To evaluate the quality of the regression model, the Hosmer–Lemeshow goodness-of-fit test and Nagelkerke R² test were used. A *p*-value < 0.05 was considered statistically significant. The regression model was also tested for multicollinearity. The statistical analyses were conducted using IBM SPSS version 26 (IBM Corp: Armonk, NY, USA).

## 3. Results

The median age among the participants was 16 years (range 15–17 years). Sadness (often or always) was reported by 21.8% (17.9% of female adolescents, 26.7% of male adolescents, 28.0% of those who chose the option “other” regarding gender) (Table 1). Sleeping difficulties more often than once a week were reported by 46.1%, headache (often or always) by 16.2%, abdominal pain (often or always) by 11.7%, reduced appetite (often or always) by 13.0%, tension (often or always) by 26.4%, and concentration difficulties (often or always) by 31.4% of the participants. A majority (73.5%) of the 1489 participants perceived their family financial situation as quite good or very good.

In Table 2, the crude analysis between sadness and the independent variables for the unadjusted OR and a 95% CI is presented. In Table 3, the adjusted analysis is presented to investigate the dependent variable sadness with the independent variables adjusting for gender and socio-economic status. The analysis (Table 3) shows that sadness was related to sleeping difficulties (OR: 2.00, CI: 1.48–2.70), headache (OR: 1.58, CI: 1.11–2.24), reduced appetite (OR: 2.43, CI: 1.68–3.51), tension (OR: 2.44, CI: 1.80–3.29), and concentration difficulties (OR: 2.75, CI: 2.05–3.69). There was no statistically significant relationship between sadness and abdominal pain (OR:1.33, CI: 0.89–1.99).

## 4. Discussion

The aim of this study was to investigate sadness and other health complaints among Swedish adolescents. We report two principal findings. First, our results show that sadness and other health complaints were common among Swedish adolescents. Second, sadness was related to health complaints (headache, sleeping difficulties, reduced appetite, tension and concentration difficulties).

### 4.1. The Prevalence of Sadness and Other Health Complaints

Sadness and other health complaints are common among adolescents, which is shown in our results and the HBSC report from 2017/2018 [1]. Though, in our study, being sad was significantly less common among female adolescents than the male participants and those who did not identify as female or male. This contrasts with previous research, which has mainly shown connections with being a female adolescent [1,5]. This result is thus surprising. A possible interpretation may relate to school performance. Gender differences in school performances, where female adolescents perform better than male adolescents, are well-documented [31], and previous research has shown a relationship between negative emotions and low school performance [32]. Therefore, a possible interpretation of our result is that male adolescents experience sadness when not accomplishing goals at school, especially in this specific context where the residents, in general, are highly educated. In addition, a common interpretation of gender differences regarding emotions, is that men express emotions differently than women, and women are generally viewed as more emotional than men [33,34]. These gender differences are based on social constructions [35,36]. Additionally, men do not seek help to the same extent as women when experiencing negative emotions, which can lead to increased health problems [37] and tremendous consequences. According to statistics presented by The Public Health Agency of Sweden [38], men make up two-thirds of those who commit suicide in Sweden. Suicide accounted for nearly one-third of all deaths among young people in Sweden in 2019. A recent systematic review that investigated gender differences in suicidal behavior among adolescents and young adults showed that female adolescents were at a higher risk of suicide attempts, while male adolescents were at a higher risk of committing suicide [39]. A possible interpretation can be that male adolescents do not seek help when needed, or that they are not understood by health care professionals. Female adolescents on the other hand are expected to be emotional and may therefore not get the support they need. Thus, there is a risk that adolescents’ emotional state becomes invisible or misunderstood, and they risk not getting person-centered support that affects them in a positive direction. Additionally, the adolescents in this study who chose the answer option “other” regarding gender might identify themselves as nonbinary or transgender. A review addressing the mental health of transgender youth demonstrated that transgender youth had higher rates of depression and suicidality compared to nontransgender youth [40]. This might account for why sadness, in our study, was more common in this group.

Additionally, being sad was significantly less common among those who perceived their family financial situation as good. Previous studies have shown relationships between poor financial situation and health complaints [1], and low economic status and depressive symptoms [5]. Economic resources offer status among peers, and therefore a poorer economic situation negatively influences adolescents’ status and well-being [41]. In this study’s context, where the majority have a high standard of living, a poor perceived family financial situation might negatively influence the adolescents’ experiences and health. Additionally, experiences of differing from others can lead to feelings of loneliness, and it is well-documented that loneliness is related to sadness and other depressive symptoms [42]. Thus, a low socio-economic status might affect adolescents’ negative emotions.

Adolescents’ health complaints risk negatively affecting their present well-being, but in addition, they have been shown to have a severe impact on health in adulthood. A recent long-term, community-based, follow-up study in Sweden reported that health complaints in adolescence were a predictor for severe mental illness in adulthood [43]. Adolescents’ well-being is thus important to consider both present and future perspectives.

### 4.2. The Relationship between Sadness and Other Health Complaints

A possible interpretation of the relationship between sadness and other health complaints may be an embodiment of emotions. Theories of embodied emotions can be seen as a critique of the dualistic body–mind discourse that separates the body from the mind or physical symptoms from emotions. This dualistic view has had a great impact on the treatment offered. Different understandings of how emotions arise and are expressed lead to different treatment and care [22]. The understanding of emotions as normal or abnormal is another aspect that influences which treatment and support are offered. Since sadness is both a common emotion that people repeatedly experience during life, as well as one of the main symptoms of the diagnosis of major depressive disorder, there is a risk of medicating a normal emotional state. This risk has been problematized in research [6]. By pathologizing a normal emotional state, or by not taking embodied emotions into consideration, there is a risk of offering treatment or care that is inappropriate and ineffective. This might lead to a deterioration in the health and well-being of adolescents. Emotions are connected to physical processes in the body, and treatment and support must therefore be polyperspectival [22]. By using different approaches and combining them, health care professionals can influence the circular causalities between psychological and biological processes. Therefore, the relationship between sadness and health complaints that is shown in our study is important to take into account when health care professionals meet adolescents.

Lastly, the bivariate analysis in this study showed a relationship between the variables sadness and abdominal pain, but in the multivariate analysis there was no statistically significant relationship between these variables. Previous research has shown a relationship between depressive symptoms and abdominal pain, especially among female adolescents [44], but research has also shown that abdominal pain related to menstruation is common among women [45]. The female adolescents in this study, who experienced less sadness than the male adolescents, may have experienced abdominal pain linked to menstruation, which might have affected the result.

### 4.3. Strengths and Limitations

A strength of this study is that the questions can be seen as well-proven since they have been used in HBSC surveys for several decades and have been psychometrically evaluated [30]. Another strength is the relatively high response rate and a roughly equal number of responders regarding gender. This might make the findings transferable to similar populations. The sample size is considered adequate since it was more than 10 times the number of included variables [28]. However, this study is cross-sectional and therefore it is not possible to conclude causality. Additionally, the questionnaire only contained sociodemographic data such as gender and self-rated financial situation, and it has not been possible to investigate relationships with other sociodemographic characteristics. Earlier research has shown that sadness is related to depressive symptoms and anxiety. It can be seen as a limitation that those aspects were not included in this study. However, the aim of this study was to investigate the relationship between sadness and more general and subtle health complaints that can be signs of embodied emotions, such as sleeping difficulties, headache, abdominal pain, reduced appetite, tension and concentration difficulties. Thus, other mental health status variables were not included in this study. Another limitation in this study is that the questions in the web-survey were single-item. Therefore, the responders could possibly misinterpret the meaning of the items due to ambiguity. Multiple-item scales might have been more successful in covering the full range of the phenomena, but there are also studies among adolescents which suggest the opposite [46]. Additionally, a limitation associated with self-reported data is that the answers, can vary from day to day due to internal and external circumstances. Additionally, the adolescents’ voluntariness can be questioned as the survey was distributed during school hours and might have been viewed as mandatory by the adolescents. A challenge in research among adolescents is that they are minors and thus particularly vulnerable. Ethical considerations are therefore important, and the study’s approval by the Regional Ethics Review Board in Lund, Sweden, was a prerequisite for the implementation of the study.

## 5. Conclusions

Our study showed that sadness and other health complaints are common among adolescents, and that sadness is related to health complaints. When adolescents express sadness or other health complaints, it is important to widen the understanding and reflect on what these complaints are an expression of, and take into account the body as physically and psychologically intertwined. This might entail person-centered support that fits the adolescent’s individual needs, which hopefully leads to improved well-being. The knowledge generated here can also be useful in preventive work. Research in the field is focusing primarily on mental illness, while mental health promotion among adolescents has not been studied to the same extent [47]. When interpreting sadness and other health complaints through embodiment, existential aspects are highlighted. Nevertheless, there is a lack of in-depth research focusing the existential aspects of being a young person. Therefore, research that profoundly highlights adolescents’ existential health is also needed.

## Figures and Tables

**Table 1:** Bivariate analysis of sadness and other factors among Swedish adolescents aged 15–17 years (*n* = 1489).

Factors	Total *n* (%)	Sadness Often or Always *n* = 325 (21.8%) *n* (%)	Sadness Never, Rarely or Sometimes *n* = 1164 (78.2%) *n* (%)	ꭓ²	*p*-Value
**Gender**				17.5	<0.0001
Male	643 (43.2)	172 (52.9)	471 (40.5)		
Female	821 (55.1)	146 (44.9)	675 (58.0)		
Other	25 (1.7)	7 (2.2)	18 (1.5)		
**Perceived family financial situation**				14.7	<0.0001
Average/ not very good/not good at all	394 (26.5)	113 (34.8)	281 (24.1)		
Very good/quite good	1095 (73.5)	212 (65.2)	883 (75.9)		
**Sleeping difficulties**				104.5	<0.0001
≥ Once a week	686 (46.1)	231 (71.1)	455 (39.1)		
< Once a week	803 (53.9)	94 (28.9)	709 (60.9)		
**Headache**				70.8	<0.0001
Often/always	241 (16.2)	102 (31.4)	139 (11.9)		
Never/rarely/sometimes	1248 (83.8)	223 (68.6)	1025 (88.1)		
**Abdominal pain**				55.1	<0.0001
Often/always	174 (11.7)	76 (23.4)	98 (8.4)		
Never/rarely/sometimes	1315 (88.3)	249 (76.6)	1066 (91.6)		
**Reduced appetite**				133.6	<0.0001
Often/always	193 (13.0)	104 (32.0)	89 (7.6)		
Never/rarely/sometimes	1296 (87.0)	221 (68.0)	1075 (92.4)		
**Tension**				161.3	<0.0001
Often/always	393 (26.4)	150 (46.2)	218 (18.7)		
Never/rarely/sometimes	1096 (73.6)	175 (53.8)	946 (81.3)		
**Concentration difficulties**				167.8	<0.0001
Often/always	468 (31.4)	198 (60.9)	270 (23.2)		
Never/rarely/sometimes	1021 (68.6)	127 (39.1)	894 (76.8)		

ꭓ² = Pearson’s chi-squared value.

**Table 2:** Logistic regression analysis of factors related to sadness (often or always) among Swedish adolescents aged 15–17 years (*n* = 1489).

Independent Variables	OR	(95% Cl for OR)	*p-*Value
Sleeping difficulties every week	2.04	(1.52–2.76)	<0.0001
Headache often or always	1.56	(1.20–2.22)	0.013
Abdominal pain often or always	1.27	(0.85–1.90)	0.238
Reduced appetite often or always	2.56	(1.77–3.69)	<0.0001
Tension often or always	2.47	(1.83–3.34)	<0.0001
Concentration difficulties often or always	2.82	(2.10–3.77)	<0.0001

OR = odds ratio. Cl = confidence interval. Hosmer–Lemeshow goodness-of-fit test, *p* = 0.067; Nagelkerke R² = 0.291. There were no signs of multicollinearity (tolerance ˃ 0.8).

**Table 3:** Logistic regression analysis of factors related to sadness (often or always) among Swedish adolescents aged 15–17 years adjusted by gender and economic status (*n* = 1489).

Independent Variables	OR ^1^	(95% Cl for OR)	*p-*Value
Sleeping difficulties every week	2.00	(1.48–2.70)	<0.0001
Headache often or always	1.58	(1.11–2.24)	0.011
Abdominal pain often or always	1.33	(0.89–1.99)	0.168
Reduced appetite often or always	1.43	(1.68–3.51)	<0.0001
Tension often or always	2.44	(1.80–3.29)	<0.0001
Concentration difficulties often or always	2.75	(2.05–3.69)	<0.0001

OR = odds ratio. Cl = confidence interval. ^1^ = Adjusted for gender and economic status. Hosmer–Lemeshow goodness-of-fit test, *p* = 0.724; Nagelkerke R² = 0.294. There were no signs of multicollinearity (tolerance ˃ 0.8).

## Data Availability

The data presented in this study are available on request from the corresponding author. The data are not yet publicly available due to work in progress.

## Appendix A. Description of the Included Variables

The survey included questions about the participants’ gender (“female”, “male”, or “other”), perceived family financial situation (5-point Likert scale ranging from “very good” to “very bad”), sleeping difficulties (3-point Likert scale ranging from “<1 time/week” to “≥3 times/week”), and sadness, headache, abdominal pain, reduced appetite, tension, concentration difficulties (5-point Likert scales ranging from “never” to “always”). The dichotomized variables used in the analysis were: sadness (never/rarely/sometimes = 0; often/always = 1), sleeping difficulties (<1 time/week = 0; 1–2 times/week/≥3 times/week = 1), headache (never/rarely/sometimes = 0; often/always = 1) abdominal pain (never/rarely/sometimes = 0; often/always = 1), reduced appetite (never/rarely/sometimes = 0; often/always = 1), tension (never/rarely/sometimes = 0; often/always = 1), concentration difficulties (never/rarely/sometimes = 0; often/always = 1), and perceived family financial situation (very good/quite good/ = 0; average/quite bad/very bad = 1).
